# New Insights into the Screening, Prompt Diagnosis, Management, and Prognosis of Breast Cancer

**DOI:** 10.1155/2020/8597892

**Published:** 2020-01-02

**Authors:** Philippe-Richard J. Domeyer, Theodoros N. Sergentanis

**Affiliations:** ^1^Hellenic Open University, Patras, Greece; ^2^Department of Clinical Therapeutics, “Alexandra” Hospital, Medical School, National and Kapodistrian University of Athens, Athens, Greece

Breast cancer is the most common cancer in women and the second main cause of cancer death after lung cancer. It is one of the few cancers where large-scale secondary prevention (screening) programs are of proven value. The advent of new technologies and biomarkers is currently enriching our portfolio for the prompt diagnosis of breast cancer and taking steps beyond the traditional diagnostic approaches, which have been shown to be cost-effective and are credited for the decline in mortality of breast cancer. Groups at high risk for breast cancer, such as mutation carriers, require intense and clear screening guidelines. In this special issue, we intend to cover the most recent trends regarding the aspects of screening, prompt diagnosis, management, and prognosis in the field of breast cancer.

The study by Η. Α. Szukis et al. evaluated factors associated with the initial mode of breast cancer detection in a sample of 1,322 Black women in the Women's Circle of Health Study. History of routine screening mammogram was associated with lower odds of clinical breast exam (CBE) as the initial mode of detection; on the other hand, lower body mass index, performance of breast self-examination before diagnosis, and larger tumor size were associated with increased odds of self-detection versus screening mammogram.

The paper by Y.-J. Kang et al. aimed to determine the relationship between breast density and age in the United Arab Emirates and consequently to assess if the results have implications on screening guidelines for breast cancer in this country, using a retrospective study design. The authors observed a significant inverse correlation between breast density category and age. Compared to Lebanese and Western women, the proportion of Emirati women with dense breasts was lower.

M. Leenders et al. undertook a review in the Cochrane Library, Embase, and PubMed databases up to June 2019 to assess whether extensive axillary nodal involvement can be preoperatively identified or excluded in breast cancer patients. After meticulous examination of the published studies, Leenders et al. portrayed the significant limitations of all current preoperative axillary imaging modalities in the identification/exclusion of extensive nodal involvement although negative PET/CT and negative MRI results are rather promising.

The cohort study by Y. Landman et al. examined carbon monoxide diffusing capacity (DLCO) in patients with early breast cancer without preexisting lung disease, who received anthracycline- and taxane-based adjuvant dose-dense chemotherapy (DDC). After implementation of longitudinal general linear models, the results highlighted a decrease in DLCO years after DDC, especially in older patients, a finding that points to a persistent symptomatic DLCO impairment in some cases, whereas the majority of patients recover partly.

The paper by W. Y.-Y. Wu et al. estimated the independent effects of the imaging biomarkers and other predictors on the risk of breast cancer death, using a prospective cohort study design. The application of imaging biomarkers along with other predictors classified twelve categories of risk for breast cancer death. In particular, it was shown that mammographic tumor appearance was an independent predictor of risk of breast cancer death, while controlling for conventional tumor attributes and treatment modalities. Furthermore, the casting type calcifications and the architectural distortion were positively associated with breast cancer death risk.

The paper by Z. Liu et al. evaluated the immunogenic activity of TP53 mutations in promoting breast cancer. The enrichment levels of 26 immune signatures indicating activities of diverse immune cells, functions, and pathways between TP53-mutated and TP53-wildtype BCs were compared based on two large-scale BC multiomics datasets. It was found that almost all analyzed immune signatures showed significantly higher enrichment levels in TP53-mutated BCs than in TP53-wildtype BCs, and that BC immunogenicity could be enhanced by mutant p53 via regulation of p53-mediated pathways.

I. Sušac et al. evaluated the expression and polymorphisms in the *BIRC5* gene (baculoviral IAP repeat containing 5), as well as the immunohistochemical expression of the *BIRC5*-encoded protein, survivin, in Croatian women. High survivin expression significantly correlated with negative ER status and Ki-67 expression. Interestingly, the results uncovered alleles of five BIRC5 polymorphisms (–1547C > T, –644T > C, –241C > T, 9386T > C, and 9809T > C) that were associated with younger age of breast cancer onset.

